# Risk Factors Associated With SARS-CoV-2 Seropositivity Among US Health Care Personnel

**DOI:** 10.1001/jamanetworkopen.2021.1283

**Published:** 2021-03-10

**Authors:** Jesse T. Jacob, Julia M. Baker, Scott K. Fridkin, Benjamin A. Lopman, James P. Steinberg, Robert H. Christenson, Brent King, Surbhi Leekha, Lyndsay M. O’Hara, Peter Rock, Gregory M. Schrank, Mary K. Hayden, Bala Hota, Michael Y. Lin, Brian D. Stein, Patrizio Caturegli, Aaron M. Milstone, Clare Rock, Annie Voskertchian, Sujan C. Reddy, Anthony D. Harris

**Affiliations:** 1 School of Medicine, Emory University, Atlanta, Georgia; 2 Rollins School of Public Health, Emory University, Atlanta, Georgia; 3 University of Maryland School of Medicine, Baltimore; 4 Rush University Medical Center, Chicago, Illinois; 5 Johns Hopkins University School of Medicine, Baltimore, Maryland; 6 US Centers for Disease Control and Prevention, Atlanta, Georgia

## Abstract

**Importance:**

Risks for severe acute respiratory syndrome coronavirus 2 (SARS-CoV-2) infection among health care personnel (HCP) are unclear.

**Objective:**

To evaluate the risk factors associated with SARS-CoV-2 seropositivity among HCP with the a priori hypothesis that community exposure but not health care exposure was associated with seropositivity.

**Design, Setting, and Participants:**

This cross-sectional study was conducted among volunteer HCP at 4 large health care systems in 3 US states. Sites shared deidentified data sets, including previously collected serology results, questionnaire results on community and workplace exposures at the time of serology, and 3-digit residential zip code prefix of HCP. Site-specific responses were mapped to a common metadata set. Residential weekly coronavirus disease 2019 (COVID-19) cumulative incidence was calculated from state-based COVID-19 case and census data.

**Exposures:**

Model variables included demographic (age, race, sex, ethnicity), community (known COVID-19 contact, COVID-19 cumulative incidence by 3-digit zip code prefix), and health care (workplace, job role, COVID-19 patient contact) factors.

**Main Outcome and Measures:**

The main outcome was SARS-CoV-2 seropositivity. Risk factors for seropositivity were estimated using a mixed-effects logistic regression model with a random intercept to account for clustering by site.

**Results:**

Among 24 749 HCP, most were younger than 50 years (17 233 [69.6%]), were women (19 361 [78.2%]), were White individuals (15 157 [61.2%]), and reported workplace contact with patients with COVID-19 (12 413 [50.2%]). Many HCP worked in the inpatient setting (8893 [35.9%]) and were nurses (7830 [31.6%]). Cumulative incidence of COVID-19 per 10 000 in the community up to 1 week prior to serology testing ranged from 8.2 to 275.6; 20 072 HCP (81.1%) reported no COVID-19 contact in the community. Seropositivity was 4.4% (95% CI, 4.1%-4.6%; 1080 HCP) overall. In multivariable analysis, community COVID-19 contact and community COVID-19 cumulative incidence were associated with seropositivity (community contact: adjusted odds ratio [aOR], 3.5; 95% CI, 2.9-4.1; community cumulative incidence: aOR, 1.8; 95% CI, 1.3-2.6). No assessed workplace factors were associated with seropositivity, including nurse job role (aOR, 1.1; 95% CI, 0.9-1.3), working in the emergency department (aOR, 1.0; 95% CI, 0.8-1.3), or workplace contact with patients with COVID-19 (aOR, 1.1; 95% CI, 0.9-1.3).

**Conclusions and Relevance:**

In this cross-sectional study of US HCP in 3 states, community exposures were associated with seropositivity to SARS-CoV-2, but workplace factors, including workplace role, environment, or contact with patients with known COVID-19, were not. These findings provide reassurance that current infection prevention practices in diverse health care settings are effective in preventing transmission of SARS-CoV-2 from patients to HCP.

## Key Points

**Question:**

What risk factors are associated with severe acute respiratory syndrome coronavirus 2 (SARS-CoV-2) seropositivity among health care personnel (HCP) inside and outside the workplace?

**Findings:**

In this cross-sectional study of 24 749 HCP in 3 US states, contact with an individual with known coronavirus disease 2019 (COVID-19) exposure outside the workplace was the strongest risk factor associated with SARS-CoV-2 seropositivity, along with living in a zip code with higher COVID-19 incidence. None of the assessed workplace factors were associated with seropositivity.

**Meaning:**

In this study, most risk factors associated with SARS-CoV-2 infection among HCP were outside the workplace, suggesting that current infection prevention strategies in health care are effective in preventing patient-to-HCP transmission in the workplace.

## Teaser

This cross-sectional study evaluates the risk factors associated with severe acute respiratory syndrome coronavirus 2 (SARS-CoV-2) seropositivity among health care personnel.

## Introduction

Since coronavirus disease 2019 (COVID-19) was recognized in the United States in January 2020, the risk of infection with severe acute respiratory syndrome coronavirus 2 (SARS-CoV-2) attributed to exposures in the health care workplace has been studied with conflicting results.^1,2,3,4,5,6^ It remains unclear whether certain job functions or specific workplace activities, including care for individuals with known and unknown SARS-CoV-2 positivity, increase the risk of SARS-CoV-2 infection. Furthermore, it is still unknown whether the association of individual and job-related characteristics and risk of SARS-CoV-2 infection are consistent across health care systems and over time.

To address these knowledge gaps, 4 sites in the US Centers for Disease Control and Prevention (CDC) Prevention Epicenters Program, based in academic institutions that collaborate with each other and the CDC to perform innovative infection prevention research, used previously collected serosurvey data to determine the prevalence of antibodies to SARS-CoV-2 in a large multistate study of US health care personnel (HCP). We sought to identify risk factors associated with seropositivity, including HCP demographic characteristics, work location, work exposure to patients with COVID-19, and community exposure to COVID-19 with the a priori hypothesis that community exposure but not health care exposure was associated with seropositivity. Our study builds on a prior single Prevention Epicenter cross-sectional study^7^ that explored specific workplace activities unique to that system.

## Methods

### Participating Academic Centers

We assessed SARS-CoV-2 seroprevalence in large health care systems affiliated with 4 Prevention Epicenters in Atlanta, Georgia (Emory Healthcare), Baltimore, Maryland (Johns Hopkins Medicine and University of Maryland Medical System), and Chicago, Illinois (Rush University System). The systems were primarily based in metropolitan areas and predominantly acute care hospitals, but they included more than 100 affiliated regional ambulatory locations, administrative locations, rehabilitation facilities, and skilled nursing facilities.

This study was reviewed by each site’s institutional review board and either approved or determined to be non–human participant research. At each site where this activity was deemed human participant research, a waiver for informed consent was obtained or individual informed consent was obtained. This activity was reviewed by the CDC and was conducted consistent with applicable federal law and CDC policy (eg, 45 CFR part 46; 21 CFR part 56; 42 USC §241(d); 5 USC §552a; 44 USC §3501). This study followed the Strengthening the Reporting of Observational Studies in Epidemiology (STROBE) reporting guideline.

### Serologic Testing, Data Collection, and Data Compilation

All badged HCP at each site were eligible to participate. Each site independently designed and conducted a voluntary HCP serological survey. The activities were started and implemented as part of internal quality and occupational safety assessments or research activities in each health care system. At the time of specimen collection, HCP completed a site-specific survey, including occupational activities and possible exposures to individuals with SARS-CoV-2 infection both inside and outside the workplace. Race and ethnicity were self-reported by HCP using categories defined by each site and were included in this study because of prior reports of racial disparities in SARS-CoV-2 seropositivity.^4,8,9^ Subsequently, we combined specimen and survey data collected between the weeks of April 19 and August 30, 2020.

The serological test used at each of the sites met the US Food and Drug Administration emergency use criteria and all measured immunoglobin G.^10^ The Abbott Architect assay (Abbott Laboratories), targeting the nucleocapsid protein, was used at Rush. Emory used a laboratory-developed assay,^11^ and Johns Hopkins used the QuantiVac ELISA (EUROIMMUN), both of which target the receptor binding domain of the SARS-CoV-2 spike protein.^12^ For the University of Maryland, only samples testing positive by both the VITROS assay (Ortho Clinical Diagnostics) and a laboratory-developed ELISA, both targeting the spike protein, were interpreted as positive for this study.

Sites shared a limited data set (without protected health information) for the combined analysis, including date and results of serology, 3-digit prefix of participants’ residential zip code, and survey responses. Because survey content varied between sites, questions from each survey were mapped to a common metadata set across the 4 sites. In some instances, text or ordinal values were mapped to categorical values to allow inclusion of data from all facilities, while in other instances substantial differences in the surveys prohibited definitive mapping. Mapping of key health care exposure variables are detailed in eTable 1 in the Supplement. Variables that could not be mapped definitively across facilities due to differences in question wording or response categories were excluded. The date of specimen collection was converted to the week number since January 1, 2020. Age was categorized by decade (ie, <30, 30-39, 40-49, 50-59, and ≥60 years) in the limited data set. Workplace location was categorized as emergency department, inpatient (regardless of direct care of patients with COVD-19), other locations (ambulatory, perioperative, surgical, rehabilitation or postacute care, no patient contact, worked from home), or unknown location. Because working in COVID-19–focused areas was captured differently across all sites, COVID-19 care was categorized as never providing COVID-19 care or providing any COVID-19 care. HCP were categorized into mutually exclusive job roles.

In addition to self-reported community contact with individuals with COVID-19, we assigned a zip code–based value of community exposure to COVID-19 to each HCP. Weekly data on the number of COVID-19 cases by 3-digit zip code prefix were obtained from the state departments of health in Georgia, Illinois, and Maryland. These data were combined with census population data to calculate weekly COVID-19 cumulative incidence by 3-digit zip code. Each participant in our metadata set was then assigned the value of COVID-19 cumulative incidence in their zip code of residence until 1 week prior to their test date.

### Statistical Analysis

We calculated the prevalence of SARS-CoV-2 seropositivity overall (combined across all sites) and for each site. The overall seropositivity was first modeled using unadjusted bivariate logistic regression assessing the association of each individual potential risk factor with seropositivity. We developed a mixed-effects logistic regression model, with a random intercept to account for clustering by the 4 sites, to estimate adjusted odds ratios (aORs) and 95% CIs between potential risk factors and SARS-CoV-2 seropositivity across all 4 sites. Model variables included demographic characteristics (sex, age group, race, ethnicity), community contact, COVID-19 cumulative incidence (base-10 logarithm of the COVID-19 cumulative incidence), and occupational factors (job role, workplace location, any contact with a patient with COVID-19).

Three sensitivity analyses were performed. We chose to include a random intercept in our main model to produce effect estimates representing the association between workplace factors and seropositivity across all 4 health care systems combined. A sensitivity analysis using logistic regression without a random intercept was performed and compared with the main model to determine whether including the random intercept improved model fit. A second sensitivity analysis included a time variable (month of test) in the model to account for monthly variations in unmeasured confounders. Lastly, we applied our main model to a subset of data excluding Emory to assess whether key risk factors identified in a detailed Emory-only analysis^7^ remained risk factors in this larger analysis. Analyses were conducted in R version 4.0.2 (R Project for Statistical Computing) using the lme4 package, and the code is publicly available.^13^ No prespecified level of statistical significance was set.

## Results

**Figure 1.**
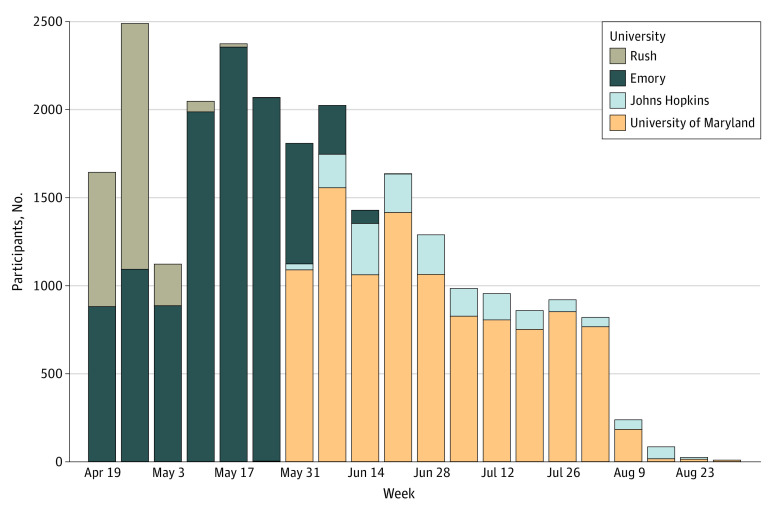
Number of Total Health Care Personnel Participating by Week and Health Care System

Among 24 952 participants from the 4 health care systems, 203 (0.8%) with indeterminate serology test results were excluded from further analysis. Of the remaining 24 749 participants, 10 275 (41.5%) were from Emory, 1626 (6.6%) from Johns Hopkins, 2470 (10.0%) from Rush, and 10 378 (41.9%) from University of Maryland. Most HCP were younger than 50 years (17 233 [69.6%]), women (19 361 [78.2%]), White individuals (15 157 [61.2%]), and non-Hispanic individuals (22 403 [90.5%]), with a large proportion of Black individuals (5117 [20.7%]). Testing volume peaked at different times at each site between April and August 2020 (Figure 1). Seropositivity for SARS-CoV-2 was 4.4% (95% CI, 4.1%-4.6%; 1080 HCP) overall and did not differ substantially by site (Table 1). Most HCP reported working predominantly in acute care hospitals (21 566 [87.1%]), with much smaller proportions working predominantly in ambulatory settings (1303 [5.3%]) or long-term care or inpatient rehabilitation facilities (630 [2.5%]); the remainder worked in administrative or other locations.

**Table 1.** Baseline Demographic and Key Characteristics of the 24 749 HCP in the Study

Characteristic	HCP, No. (%)									
	All systems		Emory		Johns Hopkins		University of Maryland		Rush	
	Total^a^	With SARS-CoV-2^b^	Total^a^	With SARS-CoV-2^b^	Total^a^	With SARS-CoV-2^b^	Total^a^	With SARS-CoV-2^b^	Total^a^	With SARS-CoV-2^b^
**Demographic and community factors**										
Total participants	24 749 (100.0)	1080 (4.4)	10 275 (100.0)	582 (5.7)	1626 (100.0)	80 (4.9)	10 378 (100.0)	319 (3.1)	2470 (100.0)	99 (4.0)
Sex										
Male	5378 (21.7)	240 (4.5)	2443 (23.8)	140 (5.7)	296 (18.2)	25 (8.4)	2104 (20.3)	56 (2.7)	535 (21.7)	19 (3.6)
Female	19 361 (78.2)	840 (4.3)	7832 (76.2)	442 (5.6)	1325 (81.5)	55 (4.2)	8274 (79.7)	263 (3.2)	1930 (78.1)	80 (4.1)
Other/unknown	10 (<0.1)	0	0	0	5 (0.3)	0	0	0	5 (0.2)	0
Age group, y										
≥60	2938 (11.9)	107 (3.6)	1238 (12.0)	59 (4.8)	146 (9.0)	11 (7.5)	1372 (13.2)	31 (2.3)	182 (7.4)	6 (3.3)
50-59	4578 (18.5)	178 (3.9)	1937 (18.9)	99 (5.1)	287 (17.7)	13 (4.5)	2045 (19.7)	58 (2.8)	309 (12.5)	8 (2.6)
40-49	5234 (21.1)	228 (4.4)	2284 (22.2)	131 (5.7)	336 (20.7)	12 (3.6)	2167 (20.9)	67 (3.1)	447 (18.1)	18 (4.0)
30-39	7454 (30.1)	328 (4.4)	3148 (30.6)	179 (5.7)	555 (34.1)	26 (4.7)	2859 (27.5)	92 (3.2)	892 (36.1)	31 (3.5)
<30	4545 (18.4)	239 (5.3)	1668 (16.2)	114 (6.8)	302 (18.6)	18 (6.0)	1935 (18.6)	71 (3.7)	640 (25.9)	36 (5.6)
Ethnicity										
Not Hispanic/Latino	22 403 (90.5)	975 (4.4)	9838 (95.7)	560 (5.7)	1551 (95.4)	77 (5.0)	8901 (85.8)	260 (2.9)	2113 (85.5)	78 (3.7)
Hispanic/Latino	1126 (4.5)	59 (5.2)	437 (4.3)	22 (5.0)	75 (4.6)	3 (4.0)	257 (2.5)	13 (5.1)	357 (14.5)	21 (5.9)
Unknown	1220 (4.9)	46 (3.8)	0	0	0	0	1220 (11.8)	46 (3.8)	0	0
Race										
White	15 157 (61.2)	499 (3.3)	5659 (55.1)	239 (4.2)	1274 (78.4)	59 (4.6)	6376 (61.4)	138 (2.2)	1848 (74.8)	63 (3.4)
American Indian or Alaska Native	105 (0.4)	5 (4.8)	33 (0.3)	2 (6.1)	1 (0.1)	0	58 (0.6)	1 (1.7)	13 (0.5)	2 (15.4)
Asian	2369 (9.6)	107 (4.5)	1253 (12.2)	65 (5.2)	179 (11.0)	9 (5.0)	679 (6.5)	19 (2.8)	258 (10.4)	14 (5.4)
Black or African American	5117 (20.7)	376 (7.3)	2986 (29.1)	246 (8.2)	108 (6.6)	8 (7.4)	1866 (18.0)	112 (6.0)	157 (6.4)	10 (6.4)
Multiracial	253 (1.0)	14 (5.5)	137 (1.3)	10 (7.3)	38 (2.3)	2 (5.3)	6 (0.1)	0	72 (2.9)	2 (2.8)
Native Hawaiian or other Pacific Islander	34 (0.1)	1 (2.9)	16 (0.2)	0	2 (0.1)	0	9 (0.1)	1 (11.1)	7 (0.3)	0
Other	702 (2.8)	25 (3.6)	0	0	24 (1.5)	2 (8.3)	563 (5.4)	15 (2.7)	115 (4.7)	8 (7.0)
Unknown	1012 (4.1)	53 (5.2)	191 (1.9)	20 (10.5)	0	0	821 (7.9)	33 (4.0)	0	0
Contact with person in community with COVID-19										
No	20 072 (81.1)	699 (3.5)	6607 (64.3)	338 (5.1)	1567 (96.4)	65 (4.1)	9735 (93.8)	228 (2.3)	2163 (87.6)	68 (3.1)
Yes	1730 (7.0)	218 (12.6)	804 (7.8)	81 (10.1)	59 (3.6)	15 (25.4)	561 (5.4)	91 (16.2)	306 (12.4)	31 (10.1)
Unknown or not reported	2947 (11.9)	163 (5.5)	2864 (27.9)	163 (5.7)	0	0	82 (0.8)	0	1	0
Cumulative community incidence of COVID-19 per 10 000, mean (range)	72.4 (8.2-275.6)		47.4 (8.2-150.4)		104.0 (45.2-273.6)		96.6 (18.8-275.6)		52.5 (10.7-147.5)	
**Workplace factors**										
Job role										
Nonclinical	5289 (21.4)	205 (3.9)	1857 (18.1)	102 (5.5)	260 (16.0)	9 (3.5)	2496 (24.1)	67 (2.7)	676 (27.4)	27 (4.0)
Nurse practitioner or physician’s assistant	1535 (6.2)	53 (3.5)	795 (7.7)	39 (4.9)	119 (7.3)	4 (3.4)	521 (5.0)	7 (1.3)	100 (4.0)	3 (3.0)
Environmental services	122 (0.5)	9 (7.4)	37 (0.4)	3 (8.1)	1 (0.1)	0	80 (0.8)	6 (7.5)	4 (0.2)	0
Nurse	7830 (31.6)	374 (4.8)	3047 (29.7)	183 (6.0)	590 (36.3)	38 (6.4)	3425 (33.0)	123 (3.6)	768 (31.1)	30 (3.9)
Other direct care personnel^c^	1914 (7.7)	105 (5.5)	1488 (14.5)	90 (6.0)	4 (0.2)	0	394 (3.8)	14 (3.6)	28 (1.1)	1 (3.6)
Other health care profressional^d^	367 (1.5)	10 (2.7)	0	0	113 (6.9)	5 (4.4)	86 (0.8)	0	168 (6.8)	5 (3.0)
Patient care technician, nursing assistant, nurse technician	1348 (5.4)	79 (5.9)	353 (3.4)	29 (8.2)	0	0	888 (8.6)	37 (4.2)	107 (4.3)	13 (12.1)
Pharmacy	325 (1.3)	10 (3.1)	0	0	35 (2.2)	4 (11.4)	215 (2.1)	6 (2.8)	75 (3.0)	0
Physician	4499 (18.2)	166 (3.7)	2116 (20.6)	97 (4.6)	388 (23.9)	18 (4.6)	1572 (15.1)	36 (2.3)	423 (17.1)	15 (3.5)
Physical, occupational, or speech therapist	483 (2.0)	17 (3.5)	0	0	76 (4.7)	1 (1.3)	339 (3.3)	13 (3.8)	68 (2.8)	3 (4.4)
Radiology technician	476 (1.9)	23 (4.8)	305 (3.0)	21 (6.9)	20 (1.2)	0	124 (1.2)	2 (1.6)	27 (1.1)	0
Respiratory therapist	399 (1.6)	18 (4.5)	115 (1.1)	7 (6.1)	20 (1.2)	1 (5.0)	238 (2.3)	8 (3.4)	26 (1.1)	2 (7.7)
Unknown	162 (0.7)	11 (6.8)	162 (1.6)	11 (6.8)	0	0	0	0	0	0
Workplace environment										
Inpatient for patients with and without COVID-19	8893 (35.9)	425 (4.8)	3576 (34.8)	218 (6.1)	713 (43.8)	37 (5.2)	3495 (33.7)	126 (3.6)	1109 (44.9)	44 (4.0)
Emergency department	2409 (9.7)	127 (5.3)	1103 (10.7)	75 (6.8)	152 (9.3)	7 (4.6)	955 (9.2)	35 (3.7)	199 (8.1)	10 (5.0)
Other	11 257 (45.5)	413 (3.7)	3821 (37.2)	192 (5.0)	761 (46.8)	36 (4.7)	5791 (55.8)	153 (2.6)	884 (35.8)	32 (3.6)
Unknown	2108 (8.5)	115 (5.5)	1775 (17.3)	97 (5.5)	0	0	144 (1.0)	5 (9.1)	278 (11.3)	13 (4.7)
Contact with patients with COVID-19										
No contact	11 435 (46.2)	448 (3.9)	5182 (50.4)	261 (5.0)	612 (37.6)	28 (4.6)	4287 (41.3)	116 (2.7)	1354 (54.8)	43 (3.2)
Any contact	12 413 (50.2)	584 (4.7)	4419 (43.0)	283 (6.4)	1014 (62.4)	52 (5.1)	6009 (57.9)	203 (3.4)	971 (39.3)	46 (4.7)
Unknown	901 (3.6)	48 (5.3)	674 (6.6)	38 (5.6)	0	0	82 (0.8)	0	145 (5.9)	10 (6.9)

Abbreviations: COVID-19, coronavirus disease 2019; HCP, health care personnel; SARS-CoV-2, severe acute respiratory syndrome coronavirus 2.

^a^ Percentages represent percentage of column.

^b^ Percentages represent percentage of row.

^c^ Other direct care personnel includes dialysis technician, phlebotomist.

^d^ Other health care professional includes laboratory technician, student, medical technologist, and other categories unable to refine.

### Setting and Participants

**Figure 2.**
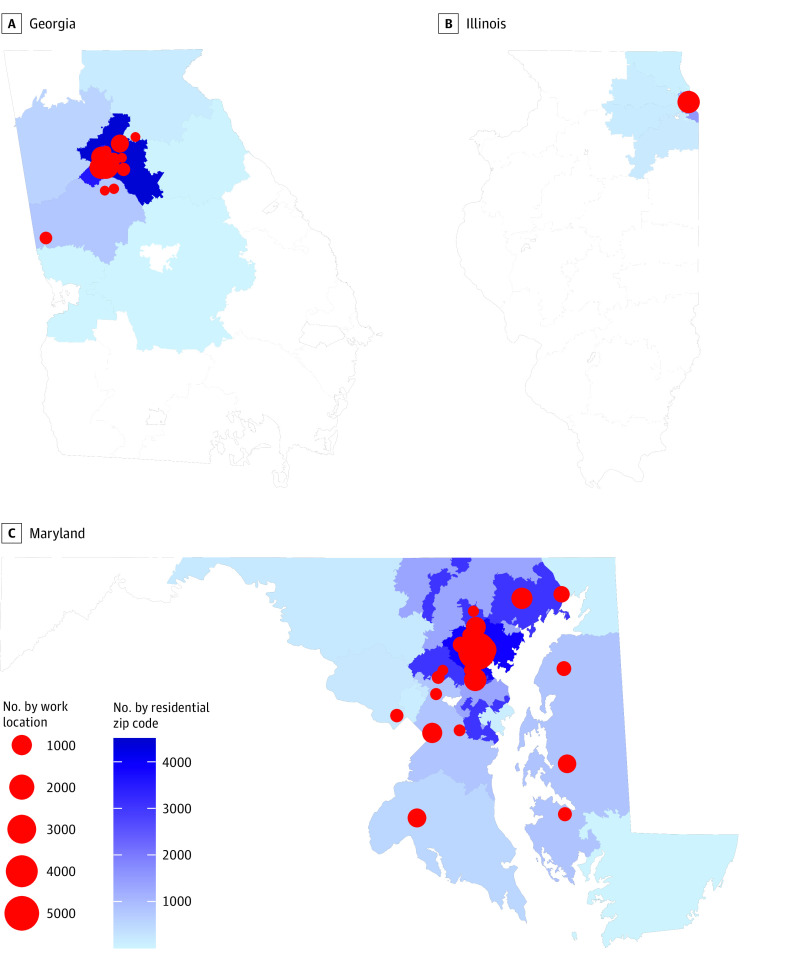
Geographic Distribution of Health Care Personnel in Each 3-Digit Zip Code and Work Locations Three-digit zip codes and work locations with fewer than 10 participants are not displayed.

HCP resided in diverse locations both inside and outside of the metropolitan areas the health care systems served, encompassing most of Georgia, Maryland, and northeastern Illinois. HCP worked in more than 100 different physical locations clustered near academic centers (Figure 2). Major infection control practices were generally consistent across sites, although there was some variation between sites and changes during the study period (eTable 3 in the Supplement).

### Community Factors and COVID-19 Incidence

Cumulative incidence of COVID-19 in the community, linked to HCP residence by 3-digit zip code 1 week prior to serology testing, ranged from 8.2 cases per 10 000 to 275.6 cases per 10 000. Community cumulative incidence during the periods of the serosurveys was generally higher for HCP at Johns Hopkins and the University of Maryland (eFigure in the Supplement), which conducted surveys later in the pandemic than Emory and Rush. Most HCP (20 072 [81.1%]) reported no known contact with a person confirmed or suspected of having COVID-19 in their community (Table 1).

### Workplace Factors

At all sites, nurse was the most common job role, with 7830 (31.6%) of the total study population (range, 3047 [29.7%] to 590 [36.3%]), followed by nonclinical staff (5289 [21.4%], eg, administrative staff, researchers, security) and physicians (4499 [18.2%]). More than one-third of HCP (8893 [35.9%]) reported working in inpatient settings (regardless of whether focused on COVID-19 care or not), 2409 (9.7%) reported working in the emergency department, and nearly half (11 257 [45.5%]) worked in other areas. Half of HCP (12 413 [50.2%]) reported caring for patients with COVID-19 or working in COVID-19–designated units.

### Regression Model Results

Of the 24 749 participants with determinate serology results, 1201 (4.9%) were excluded because of missing or out-of-state zip codes, leaving 23 548 (95.1%) for the multivariable analysis. Demographic and community factors were associated with higher odds of seropositivity among HCP (Table 2). HCP younger than 30 years had increased odds of being seropositive for SARS-CoV-2 compared with HCP 60 years of age and older (aOR, 1.3; 95% CI, 1.0-1.7); the odds of seropositivity generally decreased with increasing age. Black or African American HCP had more than twice the odds of being seropositive compared with White HCP (aOR, 2.1; 95% CI, 1.8-2.4). Sex was not associated with seropositivity.

**Table 2.** Results of Logistic Regression Analyses, Unadjusted and Adjusted, for 4 Health Care Systems With Severe Acute Respiratory Syndrome Coronavirus 2 Serology as the Outcome Variable

Factor	OR (95% CI)	
	Unadjusted (N = 24 749)^a^	Adjusted (n = 23 548)^b^
**Demographic and community factors**		
Sex		
Male	1 [Reference]	1 [Reference]
Female	1.0 (0.8-1.1)	0.8 (0.7-1.0)
Other or unknown	0.0 (NA-12.8)	0.0 (0.0-NA)
Age group, y		
≥60	1 [Reference]	1 [Reference]
50-59	1.1 (0.8-1.4)	0.9 (0.7-1.2)
40-49	1.2 (1.0-1.5)	1.0 (0.8-1.3)
30-39	1.2 (1.0-1.5)	1.1 (0.9-1.4)
<30	1.5 (1.2-1.9)	1.3 (1.0-1.7)
Ethnicity		
Not Hispanic/Latino	1 [Reference]	1 [Reference]
Hispanic/Latino	1.2 (0.9-1.6)	1.1 (0.8-1.5)
Unknown	0.9 (0.6-1.2)	0.9 (0.6-1.4)
Race		
White	1 [Reference]	1 [Reference]
American Indian or Alaska Native	1.5 (0.5-3.3)	1.4 (0.6-3.5)
Asian	1.4 (1.1-1.7)	1.2 (1.0-1.5)
Black or African American	2.3 (2.0-2.7)	2.1 (1.8-2.4)
Multiracial	1.7 (1.0-2.9)	1.3 (0.8-2.3)
Native Hawaiian or other Pacific Islander	0.9 (0.0-4.1)	0.8 (0.1-6.2)
Other	1.1 (0.7-1.6)	1.3 (0.8-2.0)
Unknown	1.6 (1.2-2.1)	1.8 (1.2-2.7)
Contact with person with COVID-19 in community		
No	1 [Reference]	1 [Reference]
Yes	4.0 (3.4-4.7)	3.5 (2.9-4.1)
Unknown or not reported	1.6 (1.4-1.9)	1.3 (1.0-1.5)
Cumulative community incidence of COVID-19 (log 10)	0.9 (0.7-1.2)	1.8 (1.3-2.6)
**Workplace factors**		
Job role		
Nonclinical	1 [Reference]	1 [Reference]
Nurse practitioner or physician’s assistant	0.9 (0.6-1.2)	0.9 (0.6-1.2)
Environmental services	2.0 (0.9-3.7)	1.5 (0.8-3.1)
Nurse	1.2 (1.0-1.5)	1.1 (0.9-1.3)
Other direct care personnel^c^	1.4 (1.1-1.8)	1.1 (0.9-1.4)
Other health care professional^d^	0.7 (0.3-1.3)	0.7 (0.4-1.3)
Patient care technician, nursing assistant, nurse technician	1.5 (1.2-2.0)	1.2 (0.9-1.6)
Pharmacy	0.8 (0.4-1.4)	0.8 (0.4-1.6)
Physician	1.0 (0.8-1.2)	0.9 (0.7-1.1)
Physical, occupational, or speech therapist	0.9 (0.5-1.5)	1.3 (0.7-2.1)
Radiology technician	1.3 (0.8-1.9)	1.0 (0.6-1.6)
Respiratory therapist	1.2 (0.7-1.9)	0.9 (0.5-1.6)
Unknown	1.8 (0.9-3.2)	0.9 (0.4-1.8)
Workplace environment		
Inpatient for patients with and without COVID-19	1 [Reference]	1 [Reference]
Emergency department	1.1 (0.9-1.4)	1.0 (0.8-1.3)
Other	0.8 (0.7-0.9)	0.9 (0.7-1.0)
Unknown	1.1 (0.9-1.4)	0.9 (0.7-1.2)
Contact with patients with COVID-19		
No contact	1 [Reference]	1 [Reference]
Any contact	1.2 (1.1-1.4)	1.1 (0.9-1.3)
Unknown	1.4 (1.0-1.9)	1.3 (0.9-1.9)

Abbreviations: COVID-19, coronavirus disease 2019; NA, not applicable; OR, odds ratio.

^a^ Crude OR between the specified factor and severe acute respiratory syndrome coronavirus 2 seropositivity using logistic regression.

^b^ OR for the association between the specified factor and severe acute respiratory syndrome coronavirus 2 seropositivity using mixed-effects logistic regression controlling for all other factors in the table and adjusting for correlation within each health care system (via inclusion of a random intercept). Random intercept had a variance of 0.07 (SD, 0.27).

^c^ Other direct care personnel includes dialysis technician or phlebotomist.

^d^ Other health care professional includes laboratory technician, student, medical technologist, other categories unable to refine.

HCP who reported having contact with a person known to have or suspected of having COVID-19 in the community had substantially increased odds of seropositivity compared with HCP with no known COVID-19 contacts outside of work (aOR, 3.5; 95% CI, 2.9-4.1). Zip code–based COVID-19 cumulative incidence (log 10) was also associated with increased odds of seropositivity (aOR, 1.8; 95% CI, 1.3-2.6).

No workplace factors were found to be associated with SARS-CoV-2 seropositivity among HCP in the multivariable regression model. Generally, any job role association with SARS-CoV-2 infection in the unadjusted analysis was attenuated in the multivariable model. Increased odds of seropositivity were estimated for HCP in environmental services (aOR, 1.5; 95% CI, 0.8-3.1) and physical, occupational, or speech therapy (aOR, 1.3; 95% CI, 0.7-2.1) compared with HCP in nonclinical roles in the multivariable model; however, the precision of these estimates was limited by the small number of HCP categorized in these roles. Notably, nurses did not have substantially increased odds of being seropositive (aOR, 1.1; 95% CI, 0.9-1.3). Neither working in the emergency department (aOR, 1.0; 95% CI, 0.8-1.3) nor providing care for patients with COVID-19 (aOR, 1.1; 95% CI, 0.9-1.3) increased the odds of seropositivity. The results from the sensitivity analyses excluding Emory data (eTable 2 in the Supplement), accounting for month of test (eTable 3 in the Supplement), and using a fixed-effects model (eTable 4 in the Supplement) were similar to those of the main model. Including a random slope in the model improved model fit when compared with the fixed-effects model (eTable 4 in the Supplement).

## Discussion

This study of more than 24 000 HCP found a low SARS-CoV-2 seroprevalence of 4.4% across multiple, geographically diverse health care systems. Most prior studies worldwide on HCP seropositivity have been limited to 1 health care system or region.^1,2,3,4,5,6,14^ Our rate of SARS-CoV-2 seropositivity was similar to that found in some health care centers^3,4^ but substantially lower than others.^6^ In our study, there was no clear association between workplace contact with patients with COVID-19 and antibody positivity, consistent with some studies^1,5^ but conflicting with others.^2,3^ We found that having community contact with COVID-19 increased the risk of being seropositive, similar to another study.^1^

While prior studies assessed community exposure primarily through self-reporting, our study found an association between cumulative incidence of COVID-19 in an HCP’s residential zip code and seropositivity across a diverse geographic area. We found that the higher the cumulative incidence of COVID-19 until the week prior to the antibody test, the higher the risk of the HCP being antibody positive. This finding aligns well with the observed association that HCP who had contact with a person with COVID-19 in the community were more likely to be antibody positive. Together, these findings suggest that exposures outside of the workplace, rather than exposures to patients with COVID-19, may be major drivers for SARS-CoV-2 infection among HCP in the United States.

A large study in Denmark that included more than 29 000 HCP found that HCP had a higher positivity rate than a contemporaneous comparison group of blood donors and that frontline HCP and HCP with more hospital exposure to COVID-19 patients had a higher risk.^2^ Another study across 7 hospitals in Denmark tested more than 25 000 HCP and found a positivity rate of 3.4%, with higher positivity among HCP taking care of patients or working in the emergency department.^3^ However, similar to our study, a large study of more than 40 000 HCP in New York found no association between work location or direct patient care and seropositivity but did not distinguish workplace and community exposures to individuals with known COVID-19.^6^

Although our study does not eliminate the possibility that workplace exposure to patients with COVID-19 increases the risk of SARS-CoV-2 infection, our findings suggest that for HCP, the risk of SARS-CoV-2 infection from community exposures may exceed the risk from patient exposures, especially considering that these findings were estimated across diverse geographic areas and health care systems. Although we were not able to assess chains of transmission in this study, it is possible that some HCP infections in the workplace were acquired during interactions in non–patient care settings, such as break rooms with other HCP with unknown SARS-CoV-2 infection. This has important implications for strategies for HCP protection. While education to HCP has focused on minimizing patient-to-HCP transmission of SARS-CoV-2, with significant effort appropriately expended to optimize patient triage, testing, and correct use of personal protective equipment, our findings suggest that additional effort is needed to prevent exposures in the community and possible workplace transmission between HCP to preserve the HCP workforce.

Recent data suggest that nurses are among the most common HCP infected with SARS-CoV-2,^15^ but this likely reflects workforce demographic characteristics given that nursing is the most common health care role.^16^ We did not find that certain job roles, including those with prolonged patient contact, such as nursing roles, had increased risk, even though nurses represented the largest portion of seropositive HCP across all job roles in our study. Importantly, these findings suggest that current infection control measures are effective for preventing SARS-CoV-2 transmission when working with patients, and HCP risk of infection may be driven by community and nonpatient care occupational exposures. Prioritizing efforts to practice optimal infection prevention in all health care facilities remains critical to keeping HCP and patients safe and may need to include assessments comparing transmission from patient-to-HCP and between HCP.

Vaccination will be important for minimizing SARS-CoV-2 infection among HCP. Despite similar levels of COVID-19 risk to non-HCP,^14^ HCP remain a priority group for vaccination for multiple reasons, including their continual potential exposures in the workplace, the importance of preserving health care capacity, and the risk of transmitting the virus from infected HCP to a large number of at-risk patients.^15^ Our findings suggest that COVID-19 vaccination and other prevention strategies targeting community transmission will also be critical to prevent SARS-CoV-2 infections in HCP.

Like previous US studies,^4,8,9^ we found that Black race was associated with seropositivity, consistent with existing health disparities in the community. Our analyses suggest that the association of Black race with SARS-CoV-2 seropositivity may be owing to existing disparities in community exposure rather than from health care–associated exposures. Surprisingly, we did not find an association between seropositivity and ethnicity, but this assessment was limited by the small number of Hispanic HCP in our study. While most previous serosurveys among HCP have not shown an association between age and SARS-CoV-2, we found that being younger than 30 years, a feature of nearly 20% of our HCP, had slightly increased risk of seropositivity. Younger HCP may be more likely to congregate in groups socially, have children in school or daycare, and have contact with other younger persons who may have fewer symptoms with infection. COVID-19 incidence was also observed to be highest among this US age group during the summer months.^17^

### Limitations

This study has important limitations. Participants were HCP who volunteered for serology testing and thus represent a convenience sample. Laboratory methods differed across sites, potentially resulting in different overall positivity rates than would have been estimated if methods had been standardized. Questionnaires used at each site were not standardized; however, we were able to successfully map many domains. Risk factors included in our multivariable model were limited to those we were able to map from all sites. For example, we were unable to assess risk associated with participation in aerosol-generating procedures because these data were only available from 3 of the 4 health care system surveys. Because infection control practices were not standardized across all sites and the practices changed during the study period (eTable 5 in the Supplement), we did not assess the association of specific infection control practices with seropositivity rates; however, we did observe similar HCP seropositivity rates despite institutional differences in personal protective equipment guidelines. In addition, we were unable to assess risk associated with exposure to an HCP with SARS-CoV-2 infection in the workplace because not all sites asked about such exposures. Furthermore, this study included predominately metropolitan HCP in acute care settings, and results may not be applicable to other health care settings, such as HCP in long-term care.

## Conclusions

Using data from across health care systems and states, this cross-sectional study found that the factors presumed to be most associated with SARS-CoV-2 infection risk among HCP, including workplace role, environment, and caring for COVID-19 patients, were not associated with increased HCP risk of SARS-CoV-2 infection. These findings provide reassurance that current infection prevention practices in similar health care systems are effective and that the largest risks may be conferred from community-based exposures. Continued efforts to minimize SARS-CoV-2 infection risk among HCP will require more detailed investigation into the specific context surrounding workplace-acquired infections among HCP and emphasis on mitigating risk outside the health care setting, including vaccine considerations and potential community-based health disparities by race among HCP.

## Notes

Supplement. **eTable 1. **Mapping of 3 Metadata Variables From Site-Specific Surveys From 4 Health Care Systems **eFigure 1. **Mean and Range for Cumulative Incidence of COVID-19 per 10 000 Among Study Participants Until 1 Week Prior to Serology Testing **eTable 2. **Results of Logistic Regression Analyses Excluding Data from Emory, With SARS-CoV-2 Serology as the Outcome Variable **eTable 3. **Results of Logistic Regression Analyses Including a Time Component (Month) in the Model, With SARS-CoV-2 Serology as the Outcome Variable **eTable 4. **Results of Logistic Regression Analyses Excluding the Random Intercept Component, With SARS-CoV-2 Serology as the Outcome Variable **eTable 5. **Infection Prevention Practices for Health Care Personnel (HCP) at the 4 Participating Health Care Systems Before (Cells Shaded Yellow) and During or After (Cells Shaded Blue) the Start of Seroprevalence Testing in HCP at That Site
