# Supplementary material for: Risk Factors Associated With SARS-CoV-2 Seropositivity Among US Health Care Personnel
Source: JAMA Netw Open. 2021 Mar 10;4(3):e211283. doi: 10.1001/jamanetworkopen.2021.1283 (PMC7948059; doi:10.1001/jamanetworkopen.2021.1283)
Supplement: Supplement. — eTable 1. Mapping of 3 Metadata Variables From Site-Specific Surveys From 4 Health Care Systems eFigure 1. Mean and Range for Cumulative Incidence of COVID-19 per 10 000 Among Study Participants Until 1 Week Prior to Serology Testing eTable 2. Results of Logistic Regression Analyses Excluding Data from Emory, With SARS-CoV-2 Serology as the Outcome Variable eTable 3. Results of Logistic Regression Analyses Including a Time Component (Month) in the Model, With SARS-CoV-2 Serology as the Outcome Variable eTable 4. Results of Logistic Regression Analyses Excluding the Random Intercept Component, With SARS-CoV-2 Serology as the Outcome Variable eTable 5. Infection Prevention Practices for Health Care Personnel (HCP) at the 4 Participating Health Care Systems Before (Cells Shaded Yellow) and During or After (Cells Shaded Blue) the Start of Seroprevalence Testing in HCP at That Site [file jamanetwopen-e211283-s001.pdf]

Supplemental Online Content

Jacob JT, Baker JM, Fridkin SK, et al. Risk factors associated with SARS-CoV-2 seropositivity among US health care personnel. *JAMA Netw Open*. 2021;4(3):e211283. doi:10.1001/jamanetworkopen.2021.1283

eTable 1. Mapping of 3 Metadata Variables From Site-Specific Surveys From 4 Health Care Systems
eFigure 1. Mean and Range for Cumulative Incidence of COVID-19 per 10 000 Among Study Participants Until 1 Week Prior to Serology Testing
eTable 2. Results of Logistic Regression Analyses Excluding Data from Emory, With SARS-CoV-2 Serology as the Outcome Variable
eTable 3. Results of Logistic Regression Analyses Including a Time Component (Month) in the Model, With SARS-CoV-2 Serology as the Outcome Variable
eTable 4. Results of Logistic Regression Analyses Excluding the Random Intercept Component, With SARS-CoV-2 Serology as the Outcome Variable
eTable 5. Infection Prevention Practices for Health Care Personnel (HCP) at the 4 Participating Health Care Systems Before (Cells Shaded Yellow) and During or After (Cells Shaded Blue) the Start of Seroprevalence Testing in HCP at That Site

This supplemental material has been provided by the authors to give readers additional information about their work.

© 2021 Jacob JT et al. *JAMA Network Open.*

eTable 1. Mapping of 3 Metadata Variables From Site-Specific Surveys From 4 Health Care Systems

Metadata Variable	Site	Site-Specific Survey Question	Site-Specific Survey Response Options	Metadata Variable Categorization from Site-Specific Survey Response Options
COVID-19 positive patient contact	Emory	A) Where do/did you spend the most time at work in the hospital during the COVID-19 pandemic? (may check more than one box if you split your time equally)<br><br>B) While at work, did/do you provide direct patient care for any patients known (confirmed) to be positive for COVID-19, regardless of location in the facility?	A) Check boxes 13 options (all that apply):<br>1. Med/surg- focused on COVID patients<br>2. Med/surg -not focused on COVID patients<br>3. ICU- focused on COVID patients<br>4. ICU- not focused on COVID patients<br>5. OR/peri-op<br>6. Other procedure area<br>7. ED<br>8. Other hospital area<br>9. Clinic- focused on COVID patients<br>10. Clinic- not focused on COVID patients<br>11. Non-clinical area of hospital/clinic<br>12. Not in hospital/clinic<br>13. Work from home<br><br>B) Option buttons (mutually exclusive categories):<br>1. Yes<br>2. No<br>3. Not sure	Any contact: A1, A3, A7, A9 and/or B1<br>No contact: A2, A4-6, A8, A10-13 and B2<br>Unknown: A missing and B3
	Johns Hopkins	Have you worked in any of the following locations since February 1, 2020? (Select all that apply)	Check boxes 4 options<br>1. Biomode [negative pressure] unit with COVID patients<br>2. Non-biomode unit with COVID patients<br>3. ED<br>4. I have not worked in any of these locations	Any contact: 1-3<br>No contact: 4<br>Unknown: N/A
	Rush	Estimate the total number of COVID-19 positive patients you have cared for	Free text	Any contact: Free text > 0<br>No contact: "None" or similar<br>Unknown: "Unknown" or similar
	University of Maryland	At work, how many patients with COVID-19 have you had direct contact with?	1. No patient contact<br>2. 1-10<br>3. 11-20<br>4. More than 20	Any contact: 2-4<br>No contact: 1<br>Unknown: N/A

© 2021 Jacob JT et al. *JAMA Network Open.*

eTable 1. Mapping of 3 Metadata Variables From Site-Specific Surveys From 4 Health Care Systems (continued)

Metadata Variable	Site	Site-Specific Survey Question	Site-Specific Survey Response Options	Metadata Variable Categorization from Site-Specific Survey Response Options
Contact with COVID-19 positive person in community	Emory	Since March 1, 2020, did you have close contact (> 10 minutes face to face) with someone with suspected or confirmed COVID-19 outside of work?	1. Yes exposed to someone with COVD-19<br>2. Yes<br>3. No<br>4. Not sure	Yes: 1, 2<br>No: 3<br>Unknown: 4
	Johns Hopkins	While not wearing a mask, have you been exposed to (select all that apply):	1. COVID positive household contact<br>2. COVID positive non-work friend<br>3. COVID positive patient<br>4. COVID positive hospital staff member<br>5. None	Yes: 1, 2<br>No: 3-5<br>Unknown: N/A
	Rush	How many COVID-19 proven or suspected individuals have you had close contact with outside of your workplace?	Free text	Yes: Free text > 0<br>No: "None" or similar<br>Unknown: "Unknown" or similar
	University of Maryland	Have you had any close contact outside of work with someone who tested positive for COVID-19?	1. Yes<br>2. No	Yes: 1<br>No: 2<br>Unknown: N/A

© 2021 Jacob JT et al. *JAMA Network Open.*

eTable 1. Mapping of 3 Metadata Variables From Site-Specific Surveys From 4 Health Care Systems (continued)

Metadata Variable	Site	Site-Specific Survey Question	Site-Specific Survey Response Options	Metadata Variable Categorization from Site-Specific Survey Response Options
Work environment	Emory	Where do/did you spend the most time at work in the hospital during the COVID-19 pandemic? (may check more than one box if you split your time equally)	1. Med/surg- focused on COVID patients<br>2. Med/surg-not focused on COVID patients<br>3. ICU- focused on COVID patients<br>4. ICU- not focused on COVID patients<br>5. OR/peri-op<br>6. Other procedure area<br>7. ED<br>8. Other hospital area<br>9. Clinic- focused on COVID patients<br>10. Clinic- not focused on COVID patients<br>11. Non-clinical area of hospital/clinic<br>12. Not in hospital/clinic<br>13. Work from home	Assigned to one location based on the following hierarchy: ED/emergency, inpatient, other, unknown<br><br>ED/emergency: 7<br>Inpatient (COVID-19/non-COVID-19): 1-4<br>Other: 5, 6, 8-13<br>Unknown: N/A
	Johns Hopkins	Have you worked in any of the following locations since February 1, 2020? (Select all that apply)	1. Biomode [negative pressure] unit with COVID patients<br>2. Non-biomode unit with COVID patients<br>3. ED<br>4. I have not worked in any of these locations	Assigned to one location based on the following hierarchy: ED/emergency, inpatient, other, unknown<br><br>ED/emergency: 3<br>Inpatient (COVID-19/non-COVID-19): 1, 2<br>Other: N/A<br>Unknown: 4
	Rush	Please identify the COVID-19 units in which you worked:	1. ED<br>2. ward<br>3. ICU<br>4. COVID-19 testing clinic<br>5. other (free text)	Assigned to one location based on the following hierarchy: ED/emergency, inpatient, other, unknown<br><br>ED/emergency: 1<br>Inpatient (COVID-19/non-COVID-19): 2, 3<br>Other: 4<br>Unknown: 5

© 2021 Jacob JT et al. *JAMA Network Open.*

eTable 1. Mapping of 3 Metadata Variables From Site-Specific Surveys From 4 Health Care Systems (continued)

Metadata Variable	Site	Site-Specific Survey Question	Site-Specific Survey Response Options	Metadata Variable Categorization from Site-Specific Survey Response Options
Work environment	University of Maryland	In which department type do you currently most often work?	1. BCU<br>2. ICU<br>3. IMC<br>4. Med/Surg Acute<br>5. Administrative Offices (outside hospital)<br>6. Support Offices (within hospital)<br>7. Pediatric<br>8. Behavioral Health<br>9. Ambulatory<br>10. Periop/Surgical<br>11. Emergency<br>12. Rehab/Post Acute<br>13. Trauma<br>14. Clinical Research<br>15. EVS<br>16. Transport	Assigned to one location based on the following hierarchy: ED/emergency, inpatient, other, unknown<br><br>ED/emergency: 11<br>Inpatient (COVID-19/non-COVID-19): 1-4<br>Other: 5-10, 12-16<br>Unknown: N/A

ICU=intensive care unit; OR=operating room; ED=emergency department; BCU=biocontainment unit; IMC=intermediate care unit; EVS=environmental services; N/A=not applicable

© 2021 Jacob JT et al. *JAMA Network Open.*

eFigure 1. Mean and Range for Cumulative Incidence of COVID-19 per 10 000 Among Study Participants Until 1 Week Prior to Serology Testing

For a given week in the figure, the mean represents the average COVID-19 cumulative incidence values for the residential zip code among the healthcare personnel who obtained serology testing that week. Similarly, the minimum and maximum COVID-19 cumulative incidence for the residential zip code were calculated among healthcare personnel who obtained serology testing that week.

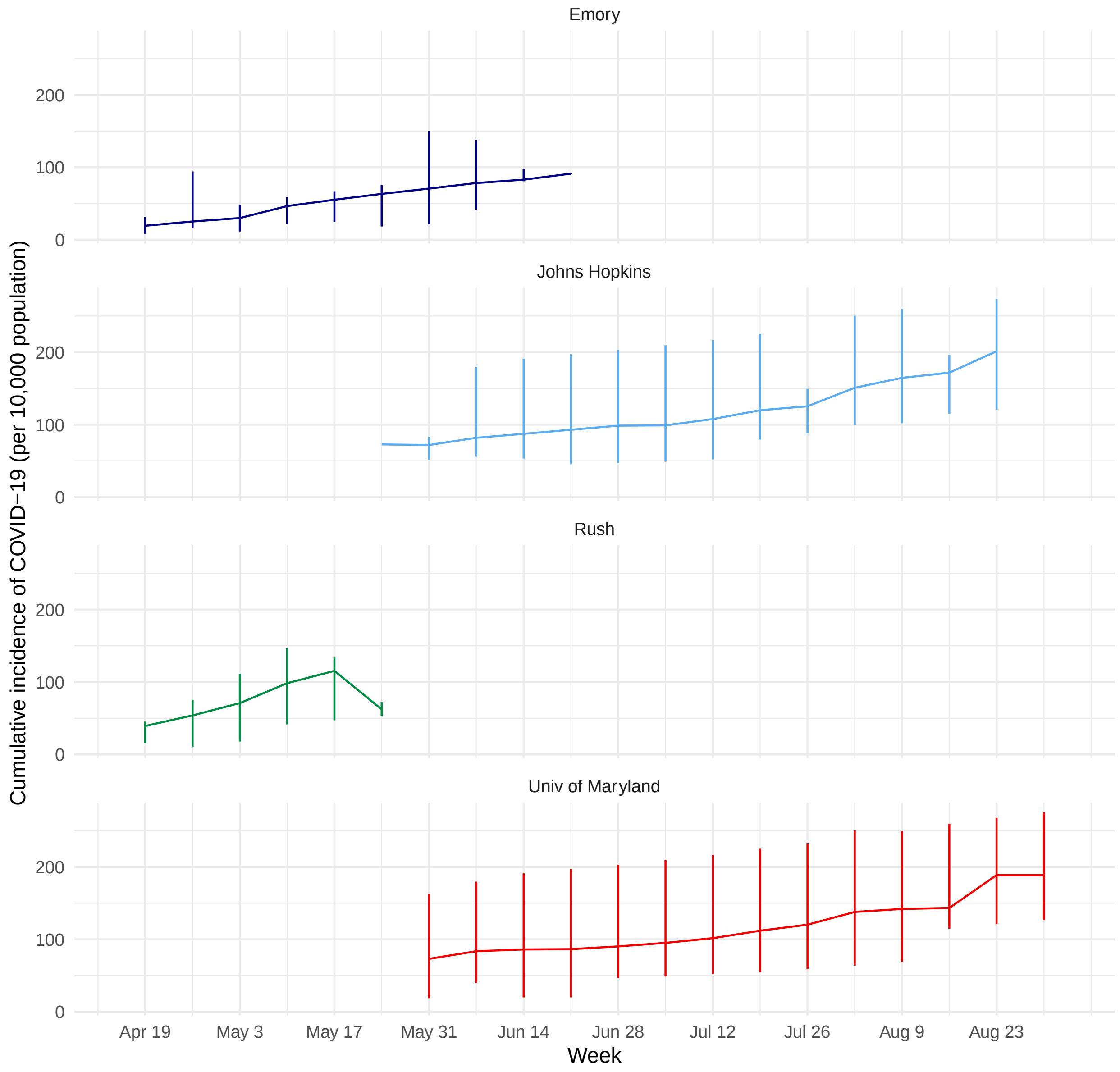

© 2021 Jacob JT et al. *JAMA Network Open.*

eTable 2. Results of Logistic Regression Analyses Excluding Data from Emory, With SARS-CoV-2 Serology as the Outcome Variable

**Factor**	**Adjusted OR[a] (95% CI)**
Total participants	13,861
**Demographic & community factors**	
Sex	
Male	1.0 (ref)
Female	0.9 (0.7-1.2)
Other/unknown	0.0 (NA)
Age group	
60+	1.0 (ref)
50-59	0.9 (0.6-1.3)
40-49	0.9 (0.6-1.3)
30-39	1.0 (0.7-1.4)
<30	1.1 (0.8-1.7)
Ethnicity	
Not Hispanic/Latino	1.0 (ref)
Hispanic/Latino	1.3 (0.9-2.0)
Unknown	1.1 (0.7-1.8)
Race	
White	1.0 (ref)
American Indian/Alaska Native	1.2 (0.4-4.1)
Asian	1.4 (1.0-1.9)
Black/African American	2.3 (1.8-2.9)
Multiracial	0.8 (0.3-2.2)
Native Hawaiian/Other Pacific Islander	2.1 (0.3-16.2)
Other	1.2 (0.8-1.9)
Unknown	1.2 (0.7-2.2)
Contact with COVID-19 positive person in community	
No	1.0 (ref)
Yes	5.9 (4.7-7.4)
Unknown	0.0 (NA)
Cumulative incidence of COVID-19 (log 10) per 10,000 population	2.7 (1.6-4.7)

© 2021 Jacob JT et al. *JAMA Network Open.*

eTable 2. Results of Logistic Regression Analyses Excluding Data from Emory, With SARS-CoV-2 Serology as the Outcome Variable (continued)

Factor	Adjusted OR[a] (95% CI)
**Workplace factors**	
Job role	
Non-clinical	1.0 (ref)
Advanced practice provider	0.5 (0.3-1.0)
Environmental services	1.7 (0.7-4.3)
Nurse	1.2 (0.9-1.6)
Other direct care provider [b]	1.2 (0.6-2.1)
Other provider [c]	0.7 (0.4-1.5)
Patient care technician, nursing assistant, nurse technician	1.3 (0.9-1.9)
Pharmacy	0.8 (0.4-1.7)
Physician	0.8 (0.5-1.1)
Physical/Occupational/Speech therapist	1.4 (0.8-2.4)
Radiology technician, x-ray, radiology	0.4 (0.1-1.7)
Respiratory therapist	1.0 (0.5-2.1)
Workplace environment	
Inpatient (COVID-19/non-COVID-19)	1.0 (ref)
Emergency department/emergency	1.2 (0.8-1.6)
Other	0.8 (0.7-1.1)
Unknown	1.1 (0.5-2.3)
COVID-19 patient contact	
No contact	1.0 (ref)
Any contact	1.2 (0.9-1.5)
Unknown	2.0 (0.7-5.5)

[a] Odds ratio for the relationship between the specified factor and SARS-CoV-2 seropositivity using mixed effects logistic regression controlling for all other factors in the table and adjusting for correlation within each healthcare system (via inclusion of a random intercept)
[b] Other direct care provider = Dialysis technician, phlebotomist
[c] Other provider = Laboratory technician, student, medical technologist, other categories unable to refine
OR= odds ratio; CI = confidence interval; ref = reference group, NA = not applicable due to small sample size

© 2021 Jacob JT et al. *JAMA Network Open.*

eTable 3. Results of Logistic Regression Analyses Including a Time Component (Month) in the Model, With SARS-CoV-2 Serology as the Outcome Variable

**Factor**	**Adjusted OR[a] (95% CI)**
Total participants	23,548
**Demographic & community factors**	
Sex	
Male	1.0 (ref)
Female	0.8 (0.7-1.0)
Other/unknown	0.0 (0.0-NA)
Age group	
60+	1.0 (ref)
50-59	0.9 (0.7-1.2)
40-49	1.0 (0.8-1.3)
30-39	1.1 (0.9-1.4)
<30	1.3 (1.0-1.7)
Ethnicity	
Not Hispanic/Latino	1.0 (ref)
Hispanic/Latino	1.1 (0.8-1.5)
Unknown	0.9 (0.6-1.4)
Race	
White	1.0 (ref)
American Indian/Alaska Native	1.4 (0.6-3.5)
Asian	1.2 (1.0-1.5)
Black/African American	2.1 (1.8-2.4)
Multiracial	1.3 (0.8-2.3)
Native Hawaiian/Other Pacific Islander	0.8 (0.1-6.2)
Other	1.3 (0.8-2.0)
Unknown	1.8 (1.2-2.7)
Contact with COVID-19 positive person in community	
No	1.0 (ref)
Yes	3.5 (2.9-4.1)
Unknown	1.3 (1.0-1.5)
Cumulative incidence of COVID-19 (log 10) per 10,000 population	1.7 (1.1-2.8)

© 2021 Jacob JT et al. *JAMA Network Open.*

eTable 3. Results of Logistic Regression Analyses Including a Time Component (Month) in the Model, With SARS-CoV-2 Serology as the Outcome Variable (continued)

Factor	Adjusted OR[a] (95% CI)
**Workplace factors**	
Job role	
Non-clinical	1.0 (ref)
Advanced practice provider	0.9 (0.6-1.2)
Environmental services	1.5 (0.8-3.1)
Nurse	1.1 (0.9-1.3)
Other direct care provider [b]	1.1 (0.8-1.4)
Other provider [c]	0.7 (0.4-1.3)
Patient care technician, nursing assistant, nurse technician	1.2 (0.9-1.6)
Pharmacy	0.8 (0.4-1.6)
Physician	0.9 (0.7-1.1)
Physical/Occupational/Speech therapist	1.3 (0.7-2.1)
Radiology technician, x-ray, radiology	1.0 (0.6-1.6)
Respiratory therapist	0.9 (0.5-1.6)
Unknown	0.9 (0.4-1.8)
Workplace environment	
Inpatient (COVID-19/non-COVID-19)	1.0 (ref)
Emergency department/emergency	1.0 (0.8-1.3)
Other	0.9 (0.7-1.0)
Unknown	0.9 (0.7-1.2)
COVID-19 patient contact	
No contact	1.0 (ref)
Any contact	1.1 (0.9-1.3)
Unknown	1.3 (0.9-1.9)
Month of test	
April	1.0 (ref)
May	1.1 (0.8-1.4)
June	1.1 (0.8-1.7)
July	1.1 (0.7-1.7)
August	1.0 (0.6-1.8)

[a] Odds ratio for the relationship between the specified factor and SARS-CoV-2 seropositivity using mixed effects logistic regression controlling for all other factors in the table and adjusting for correlation within each healthcare system (via inclusion of a random intercept)
[b] Other direct care provider = Dialysis technician, phlebotomist
[c] Other provider = Laboratory technician, student, medical technologist, other categories unable to refine
OR= odds ratio; CI = confidence interval; ref = reference group, NA = not applicable due to small sample size

© 2021 Jacob JT et al. *JAMA Network Open.*

eTable 4. Results of Logistic Regression Analyses Excluding the Random Intercept Component, With SARS-CoV-2 Serology as the Outcome Variable

**Factor**	**Adjusted OR[a] (95% CI)**
Total participants	23,548
**Demographic & community factors**	
Sex	
Male	1.0 (ref)
Female	0.8 (0.7-1.0)
Other/unknown	0.0 (NA-9.3)
Age group	
60+	1.0 (ref)
50-59	0.9 (0.7-1.2)
40-49	1.0 (0.8-1.3)
30-39	1.1 (0.9-1.4)
<30	1.3 (1.0-1.7)
Ethnicity	
Not Hispanic/Latino	1.0 (ref)
Hispanic/Latino	1.2 (0.9-1.6)
Unknown	0.7 (0.5-1.1)
Race	
White	1.0 (ref)
American Indian/Alaska Native	1.3 (0.5-3.0)
Asian	1.3 (1.0-1.6)
Black/African American	2.2 (1.9-2.6)
Multiracial	1.5 (0.8-2.5)
Native Hawaiian/Other Pacific Islander	0.8 (0.0-4.0)
Other	1.1 (0.7-1.6)
Unknown	1.9 (1.2-2.8)
Contact with COVID-19 positive person in community	
No	1.0 (ref)
Yes	3.7 (3.1-4.3)
Unknown	1.5 (1.2-1.8)
Cumulative incidence of COVID-19 (log 10) per 10,000 population	1.1 (0.8-1.4)

© 2021 Jacob JT et al. *JAMA Network Open.*

eTable 4. Results of Logistic Regression Analyses Excluding the Random Intercept Component, With SARS-CoV-2 Serology as the Outcome Variable (continued)

**Factor**	**Adjusted OR[a] (95% CI)**
**Workplace factors**	
Job role	
Non-clinical	1.0 (ref)
Advanced practice provider	0.9 (0.7-1.3)
Environmental services	1.5 (0.7-2.9)
Nurse	1.1 (0.9-1.4)
Other direct care provider [b]	1.2 (0.9-1.6)
Other provider [c]	0.8 (0.4-1.4)
Patient care technician, nursing assistant, nurse technician	1.1 (0.8-1.5)
Pharmacy	0.7 (0.3-1.4)
Physician	0.9 (0.7-1.2)
Physical/Occupational/Speech therapist	1.2 (0.7-1.9)
Radiology technician, x-ray, radiology	1.1 (0.7-1.7)
Respiratory therapist	0.9 (0.5-1.5)
Unknown	1.0 (0.5-2.0)
Workplace environment	
Inpatient (COVID-19/non-COVID-19)	1.0 (ref)
Emergency department/emergency	1.0 (0.8-1.3)
Other	0.8 (0.7-1.0)
Unknown	1.0 (0.7-1.3)
COVID-19 patient contact	
No contact	1.0 (ref)
Any contact	1.0 (0.9-1.2)
Unknown	1.2 (0.8-1.7)

[a] Odds ratio for the relationship between the specified factor and SARS-CoV-2 seropositivity using mixed effects logistic regression controlling for all other factors in the table and adjusting for correlation within each healthcare system (via inclusion of a random intercept)
[b] Other direct care provider = Dialysis technician, phlebotomist
[c] Other provider = Laboratory technician, student, medical technologist, other categories unable to refine
OR= odds ratio; CI = confidence interval; ref = reference group, NA = not applicable due to small sample size
Chi-square p-value from a likelihood ratio test comparing model fit for the random effects and fixed effect model was <0.001 indicating that the random intercept improved model fit.

© 2021 Jacob JT et al. *JAMA Network Open.*

eTable 5. Infection Prevention Practices for Health Care Personnel (HCP) at the 4 Participating Health Care Systems Before (Cells Shaded Yellow) and During or After (Cells Shaded Blue) the Start of Seroprevalence Testing in HCP at That Site

Serology start date varied by site, and the earliest started the week of 4/19/2020

Site	Enhanced hand hygiene, use of gowns, gloves for routine care of known or suspected COVID-19 patients	N95 or other respirator for routine care of known or suspected COVID-19 patients	N95 or other respirator for aerosol-generating procedure	Required universal masking of HCP	Required universal eye protection of HCP for patient encounters	Universal testing for patients on admission regardless of symptoms
Emory	Prior to 4/2020	Prior to 4/2020[a]	Prior to 4/2020	4/17/2020[b]	7/24/2020	5/3/2020
Johns Hopkins	Prior to 4/2020	Prior to 4/2020	Prior to 4/2020	4/3/2020	4/23/2020	5/13/2020
Rush	Prior to 4/2020	Prior to 4/2020[c]	Prior to 4/2020	3/27/2020	11/9/2020	N/A
University of Maryland	Prior to 4/2020	Prior to 4/2020	Prior to 4/2020	4/1/2020	4/1/2020	5/6/2020

[a] Due to contemporary public health guidance and uncertain respirator availability, procedure masks were recommended 3/20/20 to 4/1/2020.
[b] Available and suggested, but not required, starting 3/31/2020.
[c] Due to contemporary public health guidance and uncertain respirator availability, procedure masks were recommended 3/20/20 to 4/8/2020 and either a respirator or procedure mask was recommended after 4/9/2020.

© 2021 Jacob JT et al. *JAMA Network Open.*
